# Role of Fused *Mycobacterium tuberculosis* Immunogens and Adjuvants in Modern Tuberculosis Vaccines

**DOI:** 10.3389/fimmu.2014.00188

**Published:** 2014-04-23

**Authors:** Ana Paula Junqueira-Kipnis, Lázaro Moreira Marques Neto, André Kipnis

**Affiliations:** ^1^Department of Microbiology, Immunology, Pathology and Parasitology, Institute of Tropical Pathology and Public Health, Federal University of Goiás, Goiânia, Brazil

**Keywords:** fusion, adjuvant, peptides, protection

## Abstract

Several approaches have been developed to improve or replace the only available vaccine for tuberculosis (TB), BCG (Bacille Calmette Guerin). The development of subunit protein vaccines is a promising strategy because it combines specificity and safety. In addition, subunit protein vaccines can be designed to have selected immune epitopes associated with immunomodulating components to drive the appropriate immune response. However, the limited antigens present in subunit vaccines reduce their capacity to stimulate a complete immune response compared with vaccines composed of live attenuated or killed microorganisms. This deficiency can be compensated by the incorporation of adjuvants in the vaccine formulation. The fusion of adjuvants with *Mycobacterium tuberculosis* (*Mtb*) proteins or immune epitopes has the potential to become the new frontier in the TB vaccine development field. Researchers have addressed this approach by fusing the immune epitopes of their vaccines with molecules such as interleukins, lipids, lipoproteins, and immune stimulatory peptides, which have the potential to enhance the immune response. The fused molecules are being tested as subunit vaccines alone or within live attenuated vector contexts. Therefore, the objectives of this review are to discuss the association of *Mtb* fusion proteins with adjuvants; *Mtb* immunogens fused with adjuvants; and cytokine fusion with *Mtb* proteins and live recombinant vectors expressing cytokines. The incorporation of adjuvant molecules in a vaccine can be complex, and developing a stable fusion with proteins is a challenging task. Overall, the fusion of adjuvants with *Mtb* epitopes, despite the limited number of studies, is a promising field in vaccine development.

## Introduction

It is undeniable that vaccination is the best strategy available to efficiently control infectious diseases. For instance, the eradication of several infectious diseases concomitant to the lowering of morbidity and mortality rate of others can currently only be achieved by vaccination strategies. However, in the case of tuberculosis (TB), the development of a vaccine (*Mycobacterium bovis* BCG) did not have the capacity to eradicate the illness, and it persists as the second leading cause of deaths by infectious diseases, behind only AIDS (1, 2). Consequently, efforts have been made to develop vaccines that will improve or replace BCG, with the capacity to avoid infection and prevent the development of any of its disease forms and that is safe among immunocompromised individuals and capable of eliciting a protective immune response by several cellular populations (3).

Among the most promising strategies are the protein subunit vaccines that present desirable qualities for a vaccine, which are specificity, safety, and easy production (4). Protein subunit vaccines have been shown to induce a Th1 immune response, which is classically the response primarily associated with protection against TB. Such a response is characterized by the production of cytokines such as gamma interferon (IFN-γ), which is responsible for macrophage activation; tumor necrosis factor alpha (TNF-α), which is important for granuloma development and maintenance; and interleukin 2 (IL-2), which is responsible for the clonal expansion of T lymphocytes and is thus involved in immune response maintenance (5, 6). Due to these characteristics, several protein subunit vaccines are currently in advanced clinical trials (3, 7).

The selection of protein subunit vaccine components is based on the knowledge of which of the microorganism’s molecules are capable of eliciting a protective immune response (8). Therefore, due to the high level of complexity involved in the interaction between *Mycobacterium tuberculosis* (*Mtb*) and its host, the understanding of the bacteria’s immunogenic repertoire is of utmost importance for the development of an efficient vaccine. Antigens that are recognized by the host cells during active TB, when the *bacilli* are replicating, or during latent infection as well as those involved in the immunologic evasion mechanisms or the elicitation of CD4+ and CD8+ specific T cells are potential targets for immunologically controlling infections (3, 7). As the selection of potential proteins is not easy due to the vast number of MHC polymorphisms, it is necessary to select or design proteins that present promiscuous epitopes (9). Thus, the capacity of a single protein to induce an efficient immune response is inferior to other vaccine strategies (e.g., attenuated and viral vector vaccines), and their utilization is strictly associated with the use of adjuvants and immunomodulators (4, 10, 11).

Adjuvants, in the context of vaccines, are defined as components capable of enhancing and/or shaping antigen-specific immune responses (12). They can be divided into two classes: vehicles, which present vaccine antigens to the immune system in a more efficient way and control the release and storage of antigens to increase the specific immune response; immunostimulants, which affect the immune system and increase the immune responses to antigens (13). An adjuvant to be used in a vaccine against TB must have the capacity to support the generation of a robust and lasting Th1 type response. Few adjuvants are licensed to use in human vaccines, and the majority of them are poor inducers of Th1 type responses (squalene-based emulsions and aluminum-based salts). Currently, several investigations have been conducted with the objective of developing new adjuvants, many of which have searched for adjuvants that are capable of eliciting a Th1 immune response. One of these approaches is the incorporation of molecules that are capable of interaction with the pattern recognition receptors (PRRs) used by the innate immune system to recognize pathogen-associated molecular patterns (PAMPs), which are molecules or motifs that are conserved and present exclusively among pathogens (14).

**Figure 1:**
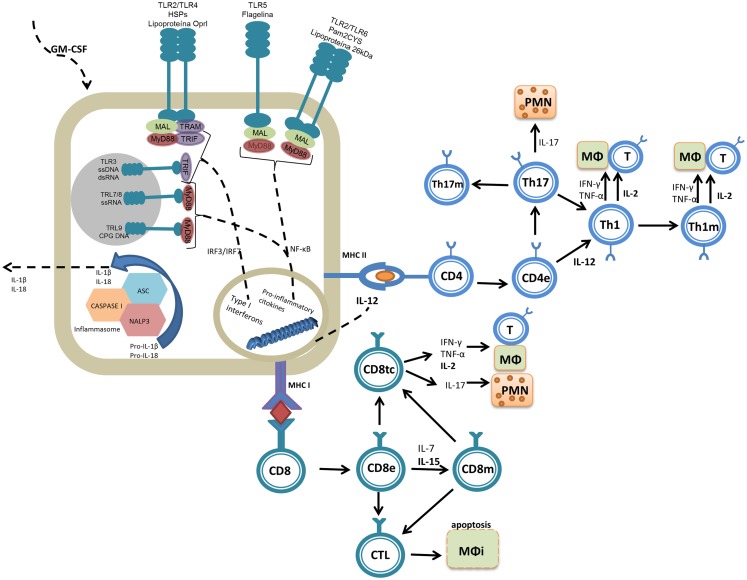
**Molecular mechanisms of the adjuvant molecules reviewed.** Adjuvants are molecules that promote inflammatory reactions, interacting with the innate immune system and assisting in the generation of adaptive immune responses. PAMPs interact primarily with TLR2 (lipoproteins and Pam2Cys) and TLR5 (flagellin). DAMPs (e.g., Hsp70) can be recognized by many molecules of the immune system, but the innate immune system interacts with Hsp70 primarily through TLR4. Adjuvants lead to the generation of intracellular signaling cascades (dependent on MyD88 and TRIF) that culminate with the production of pro-inflammatory cytokines. The cytokines then act as the third signal, aiding the development of the adaptive immune response in combination with the presentation of the fused antigen (first signal). Effector (e) and memory (m) cells are generated upon antigen presentation. Some cytokines induced by the adjuvants or used as adjuvants, such as IL-2, IL-12, IL-15, and GM-CSF, are important for activation of the protective immune response or to maintain a long-lasting immune response (memory). Among the T cell subsets induced during vaccination, Th1 cells (which produce IL-2, IFN-γ, and TNF-α) can stimulate macrophages (MΦ) and Th17 cells (which produce IL-17) activate primarily polymorphonuclear cells (PMNs), and these have been directly associated with protection against *Mtb*. Some adjuvants also induce antigen presentation via MHC-I and thus activate CD8+ T cells, which differentiate into cytotoxic cells (CD8tc) and act on infected (MΦi) or effector cells (CD8e) to secrete cytokines and differentiate into memory cells (CD8m).

Given the distinct biochemical properties of PAMPs (peptidoglycan, flagellin, lipopolysaccharide, teichoic and lipoteichoic acids, mannose residues, CpG DNA, and single-stranded RNA, among others), several types of receptors have been described [toll-like receptors (TLRs), RIG-1, NOD, and scavengers for example]. The TLR family is the most abundant and diversified and present on antigen-presenting cells (APCs) and many other cell types not related to the immune system. Signaling through TLRs can result in two possible cascades: the first is dependent on the molecule MYD88 (for myeloid differentiation factor 88) and related to TLR1, TLR2, TLR4, TLR5, TLR6, TLR7, TLR8, and TLR9; the second is dependent on TIF (TIR-domain-containing adaptor-inducing interferon-β) and is associated with TLR3 and TLR4 (15). The recognition of PAMPs by TLRs can result in the expression of co-stimulatory molecules such as CD40, CD80, and CD86 as well as the expression of pro-inflammatory cytokines (IL-1, IL-6, IL-8, IL-12, TNF-α, COX-2, and type 1 interferons) that collectively are related to the development of an adaptive immune response by both B and T lymphocytes (16) (Figure 1).

To improve the number of epitopes associated with a specific vaccine, fusions of different proteins are being developed. Vaccines based on fusion proteins have specific bacterial protein antigens comprised of proteins with better immunogenic capacities than vaccines composed of a single protein. However, this approach is not sufficient to induce a desired immune response, and several molecules with potential immunogenic properties are needed in association with fusion proteins. In this regard, the fusion of *Mtb* antigens to PAMPs (17–20), damage-associated molecular patterns (DAMPs) (21), and/or to interleukins [Table 1; Ref. (22–26)] enhances the innate immune system response, increases the capacity of the antigen to stimulate higher production of cytokines and chemokines, and elicits distinct cell populations that will aid in the defense against the bacteria (Table 1). This review covers publications that used different strategies to fuse *Mtb* antigens with adjuvants or with molecules with the capacity to interact and stimulate the immune system, addressing their immunogenicity and protection outcomes in vaccine models.

**Table 1:** **Reviewed studies that evaluated fusions of *M. tuberculosis* antigens with adjuvant molecules: molecules used and the generated immune response**.

Reference	Antigen	Adjuvant	Elicited response					
			Antibodies	Cytokines	CD4+	CD8+	Memory	Protection^a^
(18)	ESAT-6	26 kDa lipoprotein	N	IL-12p40 ↑	IFNγ ↑	N	N	=
(20)	HspX_91–110_	Pam2Cys	N	IL-6 ↑, IL-12 ↑, IFNγ ↑	CD4+ IFNγ+ ↑	N	CD44^hi^ CD62^hi^ ↑	↑
							CD44^hi^ CD62^lo^ ↑	
(17)	Ag85A	OprI lipoprotein	IgG ↑, IgG2a ↑	IL-2 ↑, IFNγ ↑, IL-10 ↑	N	N	N	=
(19)	PPE 27 kDa	Flagellin	IgG1 ↑, IgG2a ↑, IgG2b ↑	IFNγ ↑	N	N	N	N
(21)	ESAT-6	C-terminal Hsp70	IgG ↑	IFNγ ↑, IL-4 ↑	N	N	N	N
(24)	Hsp65	IL-2	IgG ↑	IFNγ ↑, IL-2 ↑	N	CTL ↑	N	=
(22)	ESAT-6	IL-2	IgG ↑	IFN-γ ↑	N	CTL ↑	N	N
(25)	ESAT-6	IL-12p70	IgG ↑, IgG1 ↑, IgG2a ↑	IFN-γ ↑	CD4+ ↑	CD8+ ↑	N	↓
(23)	Ag85B	IL-15	N	IFN-γ ↑	CD4+ IFNγ+ ↑CD4+ CD44+ ↑	CD8+ IFNγ+ ↑CD8+ CD44+ ↑	CD4+ CD44+ CD62+ ↑, CD8+ CD44+ CD62+ ↑	↑
(26)	ESAT-6	GM-CSF	IgG ↑, IgG1 ↑, IgG2a ↑^1^	IFN-γ ↑,GM-CSF ↑	CD4+ ↑	CD8+ ↑	N	N

*^a^Protection compared to BCG*.

*N, not evaluated; =, equal, ↑, higher; ↓, lower*.

## Association of *Mtb* Fusion Proteins with Adjuvants

Some protein subunit vaccines against TB that are currently in clinical trials also use adjuvant molecules that are TLR agonists. The fusion Mtb72 [a protein fusion containing the antigens Mtb32 (Rv1196) and Mtb39 (Rv0125)] uses the adjuvants AS01B™(a liposomal formulation) and AS02A™(an oil-in-water emulsion) was developed by GlaxoSmithKline (GSK). The abovementioned adjuvants are composed of MPL (3-deacylated monophosphoryl lipid A) and the detergent QS-21. MPL is a detoxified derivative of lipid A from the Gram-negative bacteria *Salmonella minnesota* R595 LPS, while QS-21 (fraction 1) is a substance purified and fractionated from the bark of the South American tree *Quillaja saponaria*. The known action of MPL is through TLR4, whereas QS-21 has no related TLR agonistic action (27). In animal studies (mice and guinea pigs), vaccination with Mtb72F and the adjuvant AS01B proved to be protective, with strong induction of antibodies (IgG1 and IgG2) and enhanced production of IFN-γ by CD4+ T cells and cytotoxic activity by CD8+ T cells. Although the vaccine formulation using AS02A induced weaker immune responses, they were able to diminish the *Mtb* bacillary load in mice. In clinical trials that compared M72 (Mtb72f with three point mutations aiming to improve antigen processing and enhance protein expression) in combination with AS01B or AS02A, both vaccine formulations were shown to be safe and immunogenic, with memory cell generation (persistency was followed for 3 years) and the production of cytokines protective against *Mtb* by several cell populations, generating similar immune responses (28–30).

Two other fusion proteins (Hybrid 4 and Hybrid 56), which are currently in clinical trials, are combined with the adjuvant IC31™. This adjuvant is made of two components, a TLR9 agonist (the oligodeoxynucleotide ODN1a) and an artificial antimicrobial cationic peptide (KLKL5KLK), which serves as a vehicle. Its mechanism of action is related to TLR activation within endosomes, and as such, IC31 is a good adjuvant for use in vaccines against intracellular microorganisms. This adjuvant was shown in several animal models to aid the skewing of the immune response toward Th1 and Th17, which is most likely associated with its adjuvant effect on dendritic cells, enhancing the expression of co-stimulatory molecules (CD80, CD86, and CD40) and the expression of IL-12p40 (31, 32).

The vaccine ID93, created by the fusion of epitopes from Rv3619, Rv1813, Rv3620, and Rv2608 and proposed to improve TB prophylaxis, includes GLA-SE [a glucopyranosyl lipid (TLR4 agonist) in a stable emulsion] as an adjuvant. Baldwin et al. (33) reported that protection was associated with strong stimulation of Th1 type immune responses, with an increase in polyfunctional cells (producing IL-2, TNF-α, and IFN-γ). This vaccine approach was shown to boost BCG protection and diminish multi-drug resistant *Mtb* infection in mice, guinea pigs, and cynomolgus monkeys (34). A peptide fusion associated with a strong adjuvant that is only mixed with the recombinant protein just before the injection might be a stimulator of the BCG immune response elicited during childhood. To select the best adjuvant to combine with ID-93-GLA, different formulations including this TLR4 agonist have been tested, including water with aluminum salts, emulsions, and liposomes. The best formulation was prepared in an aqueous nanosuspension containing alum (35). However, further studies should be conducted to best define the adjuvant associated with protective immune responses and the eradication of the *bacilli* from the host.

Although not an adjuvant currently in clinical trials, TDB (trehalose-6,6-dibehenate) has drawn attention because it is a less toxic analog of TDM (trehalose-6,6-dimycolate), a critical component of the cell wall of *Mtb*. TDB arose from changes in TDM, also known as cord factor, which is a potent inducer of Th1 type responses with restricted use in humans because of its toxicity. The effect of TDB was evaluated with the H1 fusion protein (Ag85B-ESAT-6) and shown to be a powerful aid in stimulating the cellular response of Th1 and Th17 populations as well as humoral immune responses (36, 37). The Mincle receptor (a C-type lectin) is responsible for the recognition of TDB (38) and TDM (39). It has been demonstrated that the association of Mincle–Fcrγ–Syk–CARD9 is involved in the response to TDM/TDB to generate Th1 and Th17 type immune responses in addition to activating the Nlrp3 inflammasome, inducing the production of IL-1β (40, 41).

## *Mtb* Immunogens Fused with Adjuvants

The cell wall of *Mtb* is a great source of PAMPs, as TLR2 is responsible for recognizing most of the mycobacterial lipid antigens such as lipoproteins, lipoarabinomannan (LAM), and other glycolipids. In addition to its pro-inflammatory action, TLR2 activation can result in the production of cathelicidin, a peptide with microbicidal function that also acts against intracellular bacteria (42, 43). To effectively recognize these molecules, TLR2 forms heterodimers with TLR1 and TLR6. Normally diacetylated molecules are recognized by the TLR2/TLR6 heterodimer, while triacetylated molecules (the majority are found in Gram-negative bacteria and mycobacteria) and LAM are recognized by the TLR2/TLR1 heterodimer. However, this recognition scheme is not mandatory because the recognition of lipoproteins depends both on their acetylation as well as on the peptide chain (44–46). In addition, the recognition of TLR2 agonists is also affected by accessory molecules, including CD14, CD36, lipopolysaccharide-binding protein, and others (42, 47, 48).

One of the molecules capable of interacting with TLR2 is the 26 kDa lipoprotein (Rv1411) whose recognition is related to increased production of IL-12, which in turn stimulates T lymphocytes and NK cells to produce and secrete IFN-γ, the most important inducer of reactive oxygen and nitrogen species, which are key effector molecules to kill the *bacilli* (49) (Figure 1). To take advantage of the Th1 immune response-boosting effects provided by the 26 kDa lipoprotein, this protein was fused to another immunodominant *Mtb* antigen, ESAT-6, to form the fusion protein CSU-F36. This recombinant fusion protein was produced in *Mycobacterium smegmatis*, a non-pathogenic mycobacterial species that is capable of glycosylation and acylation, allowing the molecule to be recognized as a PAMP and to correctly interact with TLR2. This protein fusion was tested in C57BL/6 mice and proved to be capable of inducing strong IL-12 p40 expression, stimulating CD4+ IFN-γ+ lymphocytes and inducing protection similar to BCG in a murine model of infection [Table 1; Ref. (18)].

Another TLR2 agonist, S-[2,3-bis(palmitoyloxy)propyl]-cysteine (Pam2Cys), has also presented favorable characteristics for a fusion vaccine against TB. Pam2Cys is a potent dendritic cell stimulator and has the ability to induce antigen cross-presentation. This synthetic molecule was derived from mycoplasma MALP-2 (macrophage-activating lipopeptide) (50). When a promiscuous peptide (capable of binding to several MHC clefts) from the 16 kDa heat shock protein (HSP) (amino acids 91–110) was fused to Pam2Cys to generate L91, it was observed that this fusion was able to induce dendritic cell maturation by enhancing the expression of the co-stimulatory molecules CD40, CD80, and CD86 and by stimulating the production of IL-6 and IL-12. The immunization of mice with L91 was capable of inducing a strong Th1 response, as evidenced by the increase of IFN-γ-producing CD4+ T cells as well as memory CD44^hi^ CD62L^hi^ (central memory) and CD44^hi^ CD62L^lo^ (effector memory) CD4+ T cells. Last, L91 was more capable than BCG of protecting vaccinated guinea pigs from *Mtb* challenge by reducing the bacterial load and the pathology [Table 1; Ref. (20)].

The outer membrane lipoprotein (OprI) of *Pseudomonas aeruginosa* can bind to TLR3/TLR4, and it has been demonstrated to be capable of supporting a strong Th1 response with the production of IFN-γ and TNF-α as well as the induction of IgG2a in a *Leishmania major* model of infection (51). *P. aeruginosa* OprI was fused to antigen 85A (Ag85A) from *Mtb* and introduced in *Escherichia coli*, which has the machinery necessary to N-acylate OprI. This fusion was used in a prime-boost strategy following vaccination with Ag85A DNA or BCG. The intranasal administration of the fusion with the DNA vaccine enhanced the immune response by potentiating the production of antibodies against Ag85A and enhancing IL-2- and IFN-γ-producing cells, but it was not able to improve its protective capacity. Similarly, the OprI-Ag85A fusion improved the Th1 response induced by BCG, but it was unable to increase its protective efficacy [Table 1; Ref. (17)].

Flagellin is another PAMP with potential to be used in fusions with *Mtb* antigens. It is recognized by TLR5, a PRR that when stimulated, induces pro-inflammatory responses as well as the maturation of APCs, boosting their capacity to activate naïve T cells to which the cells will present their antigen, thus potentiating the generation of an efficient adaptive response (52). In addition, flagellin may also be recognized by other types of PRRs, such as the Nod-like receptor family CARD 4 (NLRC4) and NIP5 (neuronal apoptosis inhibitory protein 5) (53). The protein p27, a PPE family protein from *Mtb*, was fused to the flagellin of *E. coli*. The recombinant *E. coli* expressing p27 in its flagellum was tested in BALB/c mice and compared with other vaccine strategies, including a DNA vaccine, vaccination with purified p27 and Freund’s adjuvant, and vaccination using a purified protein and CpG DNA. Immunization with recombinant bacteria induced strong splenocyte proliferation as well as higher induction of IFN-γ [Table 1; Ref. (19)].

Evidently, the fusion of *Mtb* antigens with adjuvant molecules that are agonists of TLR2 and TLR5 can boost the Th1 response. However, with the exception of L91, the fusions with TLR2 agonists were incapable of generating a protective response superior to that provided by BCG. In this regard, as described by McBride et al. (43), mice deficient for TLR2 were not more susceptible to *Mtb* when compared with wild-type mice. Another important aspect of the immune response induced by a vaccine is long-lasting protection that is provided by the generation of memory cells. McBride et al. (43) also evaluated the ability of mice that lacked TLR2 to produce memory cells against TB, and despite the large number of bacterial antigens that are agonists of this particular TLR, its absence did not prevent or hinder the generation of this cell population. However, the fusion of *Mtb* proteins with PAMPs may result in conformational changes that diminish the association with its cognate PRR, reducing the expected immunological response. Furthermore, the use of PAMPs must be considered carefully, as the immune response induced against the adjuvant fused to *Mtb* immune epitopes may result in an enhanced and deleterious immune response upon infection with *Mtb* or other pathogens expressing those PAMPs.

In the case of flagellin use in recombinant live organisms, the strategy requires that the antigen inserted in the flagellin gene does not interfere with the final molecule structure, its ability to transport flagellin, or the final flagellum assembly, which would compromise the exposure of the molecule on the cellular surface and the desired goal (52). Live vaccines, such as those including Gram-negative bacteria, have other PAMPs (LPS that interacts with TLR4, peptidoglycan recognized by TLR2, and CpG DNA recognized by TLR9) and may induce synergistic actions among PRRs such as TLR5 activation, and overstimulation can occur, inducing a deleterious inflammatory response.

Heat shock proteins are expressed both constitutively and under stress conditions in all cells and are essential for several intracellular processes such as protein transport, protection against denaturation and aggregation, and protein folding. Microbial HSPs (mHSPs) have been described as DAMPs that are highly conserved between species and potentially immunogenic, with the ability to induce the production of cytokines and chemokines, increase the expression of co-stimulatory molecules and activate APCs (particularly dendritic cells). They have also been shown to be stimulators of both T cell-mediated and humoral immune responses (54, 55).

Hsp70 from *Mtb* consists of a 44 kDa ATPase, an 18 kDa domain that binds to substrate, and a 10 kDa C-terminal fragment (56). Studies with THP-1 cells indicate that its recognition is mediated through the interaction of the C-terminal portion and the innate system, primarily the heterodimer TLR2/TLR4 and CD14 as well as CD40 and CCR5 (57). This interaction induces the production of IL-12, TNF-α, CCL5, and reactive oxygen and nitrogen species. Hsp70 is also recognized by CD8+ T lymphocytes through CD40, inducing the production of CCL3, CCL4, and CCL5 (56, 58). Based on these properties, a fusion composed of the C-terminal portion of Hsp70 (amino acids 359–610) and the immunodominant antigen ESAT-6 was made and tested in BALB/c mice in a subcutaneous immunization protocol. This fusion vaccine induced an increase in total IgG specific for ESAT-6 as well as IFN-γ production and splenocyte proliferation [Table 1; Ref. (21)].

The use of HSPs as vaccines against TB is controversial, as those proteins are able to induce pro-inflammatory and modulatory responses. For instance, Hsp70 from *Mtb* has been reported to inhibit the maturation of mouse dendritic cells *in vitro* and to modulate effects capable of inducing immune tolerance to cutaneous allografts through the induction of regulatory T lymphocytes (CD4+ CD25+ Foxp3+) (59). In addition, Hsp70 has also been shown to stimulate the production of IL-10 by peripheral blood mononuclear cells from patients with arthritis (60). Consequently, despite the capacity to induce IFN-γ production, the fusion has not been tested for its capacity to generate protection against *Mtb* infection, and the production of that cytokine alone is not sufficient to correlate with protection (61).

## Cytokine Fusion with *Mtb* Proteins and Live Recombinant Vectors Expressing Cytokines

Cytokines are molecules with biological activities that are produced by immune system cells and are responsible for cell–cell communication and the generation of a response following antigen presentation, acting as co-stimulators. Several cytokines are related to protection against *Mtb* infection, including TNF-α, IL-2, IL-6, IL-8, IL-10, and IL-17 among others, thus providing a different approach for fusion development (3, 62). The fusion of *Mtb* antigens to cytokines is aimed at not only modulating the magnitude of the response but also guiding the development of the protective immune response. However, cytokine action at the injection site is part of a complex network of signaling, and the effects of the administration of a single cytokine may not be the same as those exercised by the endogenous molecule, which acts in combination with several other molecules. Additionally, the use of recombinant cytokines can cause an imbalance of the response, and in some cases, the systemic administration of cytokines (IL-2, IL-12, TNF-α, and IFN-γ) has been associated with toxic effects to the organism. However, the short half-life of several cytokines in the circulation is also related to the inadequate adjuvant properties of some of the reported strategies (63).

IL-2 is related to the suppression of *Mtb* replication through its participation in T cell maintenance and proliferation as well as in the activation of NK and γδ T cells to produce IFN-γ. A fusion protein containing the 65 kDa HSP (Hsp65) and IL-2 was used in a vaccination scheme (DDA and MPL adjuvants) and compared to the unfused Hsp65 protein (with DDA and MPL adjuvants) or BCG. The fusion protein was shown to have a superior ability to induce the production of IFN-γ and IL-2. Vaccinated mouse splenocytes were also capable of generating superior cytotoxic activity over the P815 cell line (expressing Hsp65-hIL-12). Last, the protection conferred by the recombinant fusion protein was similar to that induced by BCG [Table 1; Ref. (24)]. Another fusion protein, comprising ESAT-6 and IL-2, was inserted in BCG. BALB/c mice vaccinated with the recombinant bacteria showed significantly greater induction of *ex vivo* splenocyte proliferation and IFN-γ production when stimulated with ESAT-6 and CFPs (culture filtrate proteins), greater production of total IgG against ESAT-6 and greater lymphocyte cytotoxic activity. However, despite the potential immunogenicity, the protection of the recombinant vaccine was not evaluated (22).

IL-12 has diverse biological functions, as it acts on several immune system cells. IL-12 plays roles in both the innate and adaptive immune responses through JAK-STAT signaling, leading to effector Th1 cell differentiation and IFN-γ production by CD4+, NK, and NKT cells in the initial stages of infection (Figure 1) (64, 65). The influence of IL-12 on CD8+ T cell differentiation has also been demonstrated through its action as the third signal (66). In addition, this cytokine acts on B cells, favoring IgG2a class switching and inhibiting IgE and IgG1 (67). A recombinant BCG containing a fusion of the IL-12p70 and ESAT-6 genes was shown to induce higher IFN-γ production than the other BCG constructs analyzed (BCG, rBCG-ESAT-6, and rBCG-IL-12), as well as higher total IgG, IgG1, and IgG2a levels. However, none of the recombinant constructs surpassed the protection induced by wild-type BCG [Table 1; Ref. (25)].

IL-15 is an important cytokine in the immune response of CD8+ T lymphocytes, as it is involved in clonal expansion, memory cell (CD44^hi^) generation, and antigen recognition, and it further acts as a T lymphocyte chemoattractant (Figure 1) (68, 69). A fusion protein was designed to explore the poor CD8+ T cell stimulation capacity of BCG (70); the fusion protein comprised Ag85B and IL-15 and was expressed in BCG [Table 1; Ref. (23)]. The recombinant BCG was shown to enhance the control of infection, inducing total memory (CD44+) CD4+ and CD8+ T cells, and to potentiate the production of IFN-γ by both CD4 and CD8 T cells. Finally, the presence of IL-15 was capable of enhancing the protection against *Mtb* when compared with rBCG-Ag85B, with lower bacillary loads in the lungs and milder pathology (23).

Granulocyte macrophage colony-stimulating factor (GM-CSF) has several described biological effects, but from the vaccination perspective, its primary effects are enhancement of the maturation, migration, and immunostimulatory properties of Langerhans, dendritic, and NK cells; increasing MHC class II expression on APCs, which plays a fundamental role in antigen presentation to CD4 T helper cells; increasing the expression of CD80, a co-stimulatory molecule that participates in T lymphocyte activation, on Langerhans giant cells *in vitro*; and inducing local inflammation at the injection site, resulting in the accumulation of neutrophils and mononuclear cells (Figure 1). An important role of GM-CSF has also been demonstrated in mice lacking GM-CSF expression, which were more susceptible to *Mtb* infection (71). In one study, GM-CSF was fused to ESAT-6, and the recombinant fusion gene was inserted into BCG, creating rBCG:GE. This rBCG was tested and compared to BCG expressing either ESAT-6 (rBCG:E) or GM-CSF (rBCG:G). rBCG:GE induced higher levels of total IgG, IgG1, and IgG2a. Mice immunized with rBCG:GE also had higher levels of specific CD4+ and CD8+ T cells 8 weeks after immunization when compared with the other recombinant BCG vaccines. Last, rBCG:GE also showed the greatest capacity to stimulate IFN-γ production by the splenocytes of immunized mice. However, no protection assay was described by the authors [Table 1; Ref. (26)].

When using cytokines as immune stimulation components in vaccines, one expects that they will enhance the immune response due to their participation in the regulation of both innate and adaptive immune responses. In the published literature, IL-2, IL-12, IL-15, and GM-CSF have been reported as potential immune response stimulators. However, only the Hsp65 antigen fusion with IL-2 was used without being in the context of the BCG vector, and in that case, it was necessary to use a dimethyl dioctadecyl ammonium bromide (DDA) and MPL emulsion as an adjuvant to generate an efficient immune response, demonstrating the inability of a single cytokine to support an antigen-elicited response. Although fusions of *Mtb* antigens with cytokines have been shown to be immunogenic and to improve the response to BCG, only one of the studies we reviewed, using IL-15/Ag85B, induced levels of protection against bacteria that were better than those induced by the current vaccine. Additionally, two studies did not evaluate the protection against *Mtb*, which prevents a more thorough assessment of the real potential of the examined fusion vaccines because there is no consensus in the scientific community of a biomarker for protection.

Among the major obstacles faced in the search for adjuvants capable of stimulating Th1 type responses is the immunotoxicity generated by some molecules. Therefore, an adjuvant should be effective in assisting the generation of protective immune responses while inducing few side effects (72). However, the balance between effectiveness and toxicity in a vaccine for TB is complex because the protective immune response against the agent (Th1 and Th17) is highly inflammatory. Side effects related to the use of adjuvants can be divided into two major groups: local and systemic effects. The most common effects are local injection site tenderness and swelling, while the more severe reactions involve the formation of abscesses and painful nodules. With regard to systemic effects, the most common reaction is a non-specific acute phase reaction, characterized by changes in plasma proteins, fever, fatigue, and anorexia, whereas severe effects may include the generation of autoimmune diseases or worsening thereof and the appearance of neurological disorders (73). Systemic side effects related to the administration of adjuvants typically prevent their use in human vaccines, and these effects usually occur due to the hyperactivation of the immune system as a result of constant exposure to adjuvant, which causes an intense production of pro-inflammatory cytokines (IL-1, IL-6, TNF-α, IFN-γ, and others). Importantly, these effects may occur after the administration of a cytokine adjuvant or after the use of a molecule and subsequent infection by a microorganism that has the same molecule in its constitution, and therefore, such effects must be considered when choosing an adjuvant for a vaccine formulation (73, 74).

## Conclusion

Several fusions of *Mtb* proteins or immunodominant epitopes have been evaluated as subunit vaccines for TB. Some adjuvants have been incorporated in the vaccine formulation without a physical association with the recombinant fusion proteins, while others have been incorporated in the backbone of the subunit vaccines. Other studies have evaluated recombinant BCG vaccines expressing both *Mtb* proteins and adjuvant molecules. The majority of the vaccine formulations were able to induce higher levels of Th1 and IgG2a responses, although not all of the vaccines discussed here presented better protection against *Mtb* than BCG.

The fusion of *Mtb* antigens with adjuvants can interfere with the induction of specific immune responses, but in most of the reviewed articles, the vaccine formulations did not offer improvements over the protection conferred by BCG. Thus, further studies are needed to develop an effective adjuvant with low toxicity to be used in vaccine formulations to control TB.

## Author Contributions

Ana Paula Junqueira-Kipnis designed the review and critically wrote and edited the manuscript. Lázaro Moreira Marques Neto critically wrote the draft. André Kipnis critically wrote the manuscript and edited the manuscript. All authors read and approved the final version of the manuscript.

## Conflict of Interest Statement

The authors declare that the research was conducted in the absence of any commercial or financial relationships that could be construed as a potential conflict of interest.
